# Impact of 3D cell culture on bone regeneration potential of mesenchymal stromal cells

**DOI:** 10.1186/s13287-020-02094-8

**Published:** 2021-01-07

**Authors:** Mesude Bicer, Graeme S. Cottrell, Darius Widera

**Affiliations:** 1 Stem Cell Biology and Regenerative Medicine Group, Reading School of Pharmacy, University of Reading, PO Box 226, Whiteknights, Reading, RG6 6AP UK; 2 Cellular and Molecular Neuroscience, School of Pharmacy, University of Reading, Reading, UK

**Keywords:** 3D scaffolds, Osteogenic differentiation, Mesenchymal stem cells, Osteoregeneration, Stem cell therapy, Bone tissue engineering

## Abstract

As populations age across the world, osteoporosis and osteoporosis-related fractures are becoming the most prevalent degenerative bone diseases. More than 75 million patients suffer from osteoporosis in the USA, the EU and Japan. Furthermore, it is anticipated that the number of patients affected by osteoporosis will increase by a third by 2050. Although conventional therapies including bisphosphonates, calcitonin and oestrogen-like drugs can be used to treat degenerative diseases of the bone, they are often associated with serious side effects including the development of oesophageal cancer, ocular inflammation, severe musculoskeletal pain and osteonecrosis of the jaw.

The use of autologous mesenchymal stromal cells/mesenchymal stem cells (MSCs) is a possible alternative therapeutic approach to tackle osteoporosis while overcoming the limitations of traditional treatment options. However, osteoporosis can cause a decrease in the numbers of MSCs, induce their senescence and lower their osteogenic differentiation potential.

Three-dimensional (3D) cell culture is an emerging technology that allows a more physiological expansion and differentiation of stem cells compared to cultivation on conventional flat systems.

This review will discuss current understanding of the effects of different 3D cell culture systems on proliferation, viability and osteogenic differentiation, as well as on the immunomodulatory and anti-inflammatory potential of MSCs.

## Background

**Fig. 1:**
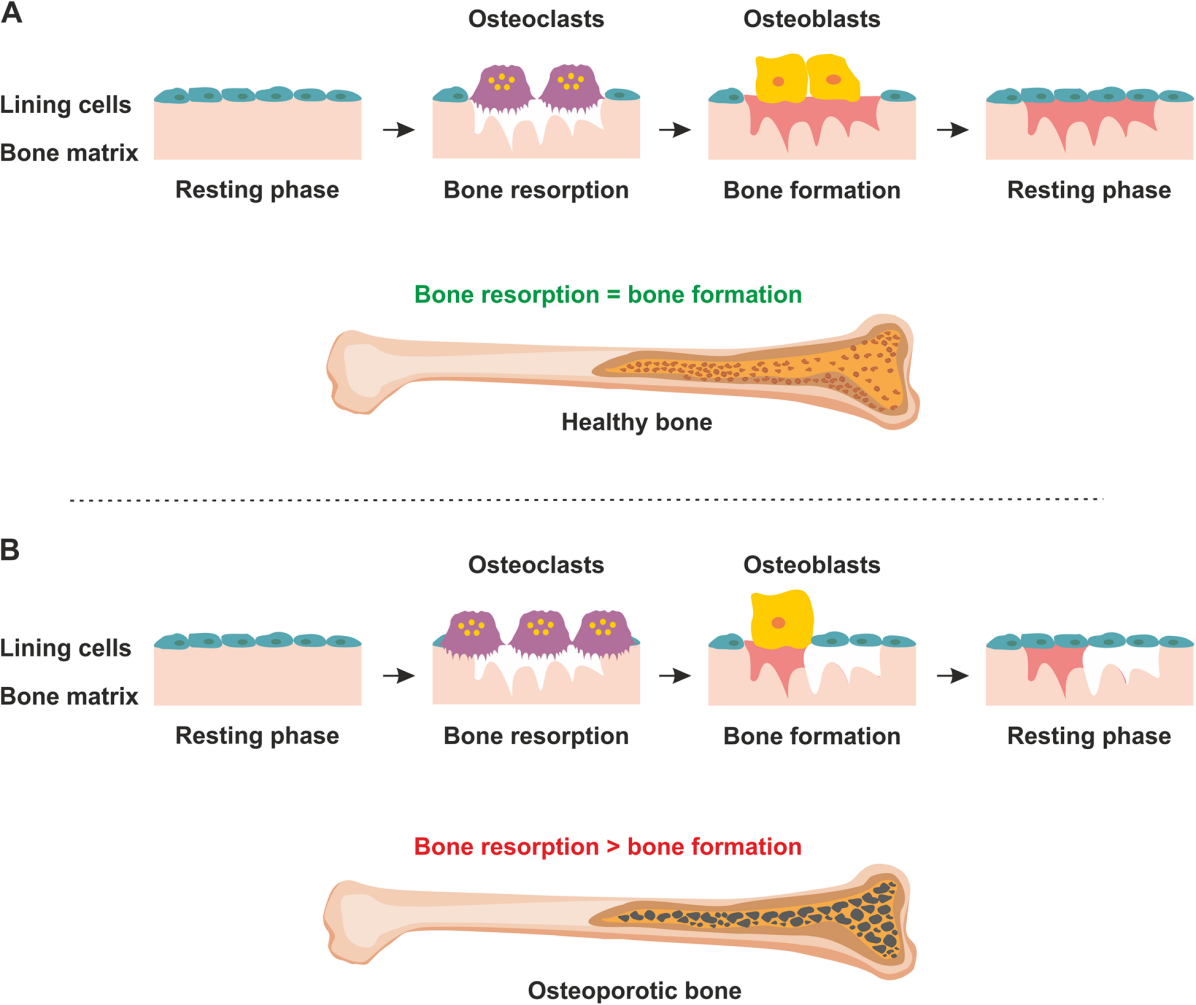
Bone remodelling cycle and bone degeneration in osteoporosis. The dynamic relationship between osteoblasts and osteoclasts is primarily regulated by a fine balance between bone formation and bone resorption. **a** In healthy bone tissue, resorption of bone following the resting phase is mediated by osteoclasts and counter-balanced by deposition of new bone material by osteoblasts. Once new bone material has been deposited, another resting phase follows. **b** With increasing age and in patients with osteoporosis, the balance shifts towards higher levels of osteoclast activation with reduced osteoblast differentiation, thereby impairing the regenerative potential of the bone and resulting in structural deterioration of the bone tissue along with reduced bone strength

Bone remodelling is a continuous cycle of degeneration and regeneration, involving osteoblasts (bone-forming cells) and osteoclasts (cells that absorb bone tissue) (Fig. 1). If the balance between bone formation and bone resorption is lost, the bone becomes vulnerable to osteoporosis [1].

Osteoporosis is characterised by low bone mass which is strongly associated with increased bone resorption combined with reduced bone regeneration [2] and mostly affects postmenopausal women [3]. In the context of bone homeostasis, oestrogen regulates osteoblast survival and suppresses cellular apoptosis [4]. The onset and development of osteoporosis are related to the life span of osteoblasts. Oestrogen plays an important role in the reduction of apoptotic gene expression in osteoblasts [5] with nuclear oestrogen receptors and androgen receptors being directly involved in the process of bone remodelling and in modulation of the levels of interleukin-6 (IL-6) [6].

In addition to its impact on osteoblasts, oestrogen has two potential roles in regulating osteoclasts. Briefly, it decreases osteoclast cell differentiation by suppressing receptor activator of nuclear factor kappa-Β ligand/macrophage colony-stimulating factor (RANKL/M-CSF) signalling [7] and indirectly blocks the production of the bone-resorbing cytokines IL-1β, IL-6, tumour necrosis factor-α (TNF-α), M-CSF and prostaglandins [8]. In addition, it inhibits bone resorption by directly inducing apoptosis of osteoclasts [9].

In addition to oestrogen, inflammatory signalling and the activity of the immune system are also involved in bone and regeneration and degeneration. In the acute phase after injury or at the onset of osteoporosis, local levels of pro-inflammatory cytokines in the bone tissue rise causing immune cell infiltration, macrophage polarisation towards the pro-inflammatory M1 phenotype and release of chemokines. This in turn induces migration of the cellular ancestors of osteoblasts, MSCs, from their niche to the bone and has a positive influence on bone regeneration [10, 11]. However, if the inflammation becomes chronic as in osteoporosis, this promotes strong and persistent activity of immune cells interfering with bone regeneration [12].

Given this complex nature of molecular and cellular mechanisms involved in osteoporosis, development of therapeutic interventions is challenging and represents a major and unmet clinical need.

## Current therapeutic options and their limitations

Prior to 2002, one of the most common therapies prescribed for osteoporosis was hormone replacement therapy (HRT). In addition to prescribing HRT for female patients with osteoporosis, postmenopausal women were advised to take oestrogen to prevent a loss of bone density [13]. In 2002, however, it was reported that HRT increases the risk of breast cancer and heart disease. As a result, less patients are now prescribed HRT [14].

In recent years, alternative approaches to treat bone degeneration have been developed. These range from systemic pharmacological approaches to surgical procedures.

Currently, the main therapeutic agents for treating osteoporosis include anti-resorptives such as HRT, selective oestrogen-receptor modulators (SERMs) and anti-RANKL antibodies. An ideal pharmacological intervention treating bone loss should suppress osteoclastic activity and enhance osteoblast-mediated bone formation [13]. A humanised monoclonal antibody (denosumab) has recently been developed that inhibits osteoclastic differentiation by preventing RANKL from binding to its receptor [13]. Long-term administration of denosumab has been shown to have positive effects not only on the prevention of fragility fracture, but also on the amelioration of joint damage in osteoporotic patients [15]. However, treatment with denosumab is linked to an increased risk of hypocalcaemia and osteonecrosis of the jaw [16].

As an alternative pharmacological treatment option, parathyroid hormone therapy (PTH) has been explored for treating severe osteoporosis in men and postmenopausal women with osteoporosis. A systematic review on the PTH for the treatment of osteoporosis revealed that PTH increases bone mineral density and reduces the risk of fractures by decreasing osteoblast apoptosis. It also enhances differentiation of osteoblasts from pre-osteoblasts, thereby inducing new bone deposition. Despite its high potential, PTH therapy has its drawbacks including side effects like headaches, injection-site tenderness and nausea in osteoporotic patients [17].

A sclerostin (SOST) antibody and interferon γ (IFNγ) also have therapeutic potential in treatment of osteoporosis [13]. The SOST antibody modulates osteoblastic activity and bone formation, by inhibiting Wnt/β-catenin signalling [18]. IFNγ can be an effective anabolic treatment for osteoporosis as in the bone micro-environment it is secreted by MSCs to promote osteogenic differentiation [19]. However, there are risks associated with local interferon therapy for bone loss, such as gastrointestinal tract symptoms [19]. Bisphosphonates (BPs) are commonly used for the treatment of osteoporosis, as they have the potential to suppress osteoclast formation [20, 21] and can break the osteoblast/osteoclast coupling. However, a drawback of BPs is that they can cause osteonecrosis of the jaw [22].

In addition to these pharmacological options, osteoporosis treatment can involve surgical procedures including autologous bone grafts, allograft implantations and free fibula vascularised grafts [23]. Even though autologous bone grafting is currently seen as the gold standard, this technique still has drawbacks in terms of a high risk of morbidity and intervention-induced inflammation [24].

Thus, safe and predictable therapies for osteoporosis are an unmet clinical need. In this respect, stem cell therapy for osteoporosis is a novel approach proposed to increase bone formation.

## MSCs as a potential stem cell therapy for bone degeneration

In light of the limitations of conventional therapies, the use of stem cells has been explored in the context of bone healing. Due to their wide availability, minimally invasive isolation procedures and their regenerative potential, MSCs are considered the most suitable stem cell type to be used in treatment of osteoporosis [25].

MSCs are fibroblast-like cells able to differentiate into mesenchymal derivatives including bone, fat and cartilage cells (see [26] for review). They can be readily obtained from various adult tissues including the bone marrow, adipose tissue and peripheral blood. Their high availability and low-risk profile made MSCs a promising stem cell type for clinical exploitation [27]. As of July 2020, over 1100 clinical trials have been registered on the ClinicalTrials.gov database with a wide indication profile including diabetes, cardiovascular disorders and musculoskeletal symptoms.

As cellular ancestors of osteoblasts, MSCs can contribute to bone regeneration either directly by integrating and differentiating into bone tissue or by indirect stimulation of endogenous regeneration processes.

Direct differentiation of rat MSCs into the osteogenic lineage was first reported by Friedenstein et al. as early as 1974 [28]. Following this discovery, MSCs from numerous species and origins have been demonstrated to undergo differentiation into osteogenic cells. Consequently, in their position paper, The International Society for Cellular Therapy Position Statement defined the ability to give rise to bone cells in vitro as one of the minimal criteria for defining MSCs [29]. In addition to in vitro differentiation, even more stringent evidence for osteogenic potential of MSCs has been provided by several in vivo studies in different small and large animal models including rats [30], sheep [31] and pigs [32].

Cumulatively, these promising in vitro and in vivo results motivated several clinical trials designed to assess the feasibility, safety and efficacy of MSCs in oral bone regeneration [33–35]. Although several individual trials suggested that transplantation of MSCs is safe and associated with improved oral bone regeneration, a more recent meta-analysis did not reveal a significant increase in vital bone mass [36]. This can be at least partly attributed to the fact that in contrast to early pre-clinical studies claiming that a high number of transplanted MSCs undergo differentiation to regenerate the bone, very few regenerative cells survive following transplantation [37]. This has been eloquently demonstrated in a recent study, where human MSCs have been transplanted into large segmental bone defects in rats [38]. Although a significantly improved healing of large segmental bone defects has been demonstrated, tracking experiments failed to conclusively demonstrate long-term survival and differentiation of MSCs in the regenerated bone.

According to the database ClinicalTrials.org, no clinical trials utilising MSC transplantation as a therapeutic option to treat osteoporosis or osteoporotic fractures have been completed as of July 2020. However, MSCs have been shown to prevent progression of osteoporosis in a mouse model of age-related osteoporosis [39]. Briefly, the authors demonstrated that a single dose of MSCs increased the bone turnover rate and reversed the age-associated reduction of bone formation compared to sham controls. These promising results indicate that MSCs may have relevance in the treatment of osteoporosis in humans.

**Fig. 2:**
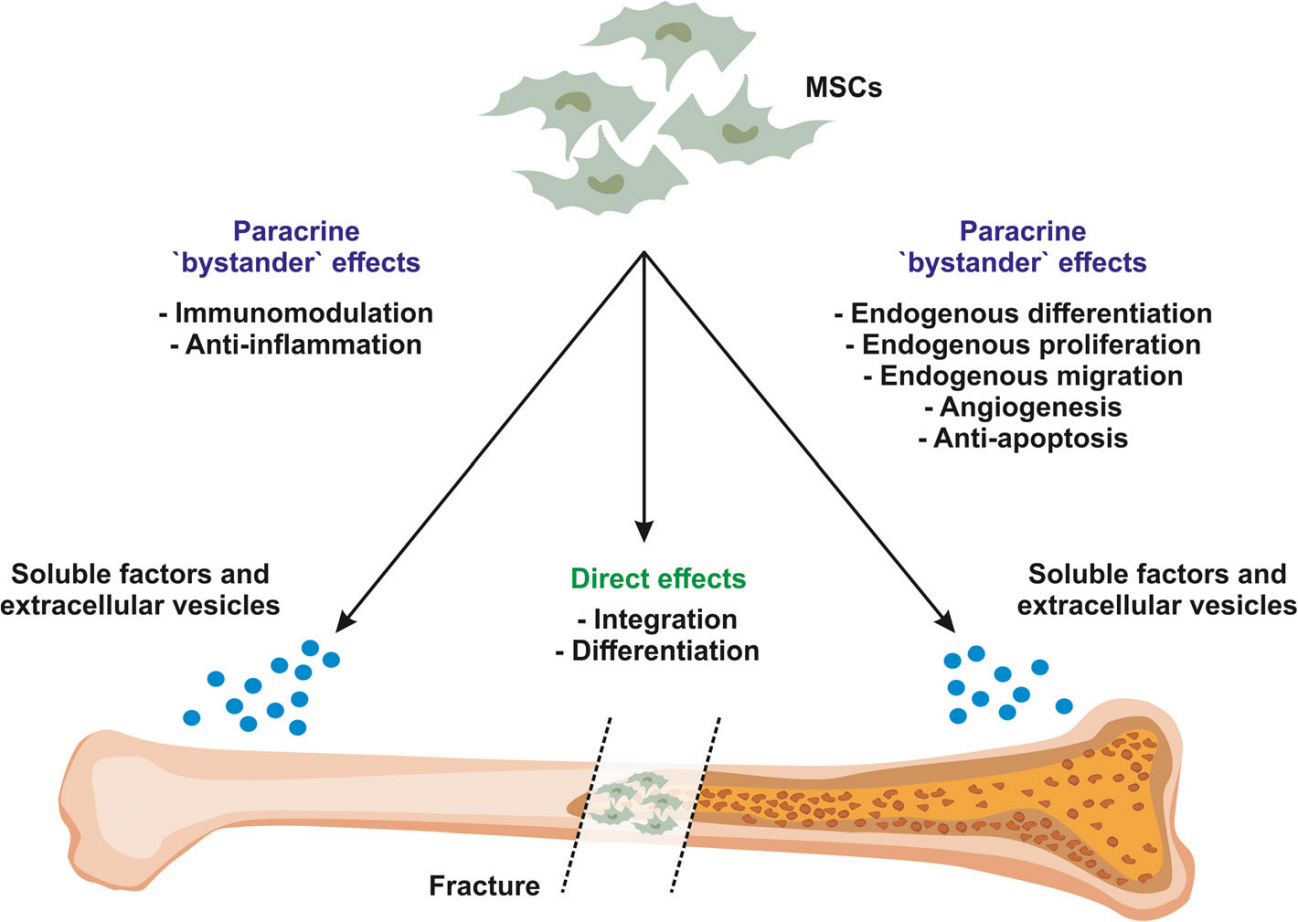
Contribution of MSCs to regeneration in bone fractures**.** In the acute phase after a bone fracture, resident or transplanted MSCs release a magnitude of paracrine factors which in turn attract immune cells and additional MSCs to the side if the injury. Paracrine actions of MSCs also include modulation of endogenous cell differentiation, proliferation and angiogenesis. In addition, they are able to integrate and undergo osteogenic differentiation thereby replacing lost bone material. Finally, in the late stage of bone regeneration, MSCs contribute to regeneration by releasing soluble and extracellular vesicle embedded immunomodulatory and anti-inflammatory factors including proteins and miRNAs

In addition to differentiation and functional integration, MSCs are widely believed to contribute to regeneration via paracrine factors, acting as ‘bystander cells’ that modulate endogenous regeneration (Fig. 2). Indeed, in light of the general consensus that the therapeutic benefit of MSCs is paracrine, Arnold I. Caplan, who first coined the term ‘mesenchymal stem cells’, recommends referring to this cell type as ‘medicinal signalling cells’ [40]. Current understanding of these ‘bystander effects’ mediated by the secretome of MSCs is that they include two main factors—immunomodulation and reduction of inflammation. In the context of immunomodulation, MSCs and their secretome can affect a wide variety of immune cells including but not limited to macrophages, dendritic cells, T cells, B cells and natural killer cells (reviewed in [41]), resulting in local immunosuppression.

Notably, the anti-inflammatory action of the MSC secretome is not limited to immune cells as it can reduce the levels of pro-inflammatory markers in other tissues including cartilage [42] and muscle tissue [43]. In addition, ‘bystander effects’ on the surrounding tissue include paracrine modulation of angiogenesis, anti-apoptosis and chemoattraction, as well as stimulation of resident cell proliferation [44].

The paracrine factors responsible for the effects introduced above are soluble factors including cytokines, growth factors and chemokines in addition to proteins and miRNAs embedded within extracellular vesicles (EVs) (reviewed in [45]). Indeed, multiple pre-clinical and clinical reports provided evidence that MSCs exert at least part of their therapeutic functions via EVs such as exosomes and microvesicles [43, 46–49]. EVs are secreted by almost all cell types under physiological and pathological situations, are anti-inflammatory and immunomodulatory and have a specific combination of proteins, lipids and microRNAs as their cargo [50, 51].

Despite the high clinical potential, MSC-based approaches to tackle osteoporosis are limited by the fact that osteoporosis has a negative impact on the number and osteogenic differentiation potential of MSCs [52–54]. This can at least partly be attributed to a shift of osteoporotic MSCs towards an adipogenic fate, which is believed to interfere with new bone formation in osteoporosis [55, 56].

An additional factor playing a role in the lower osteogenic potential of osteoporotic MSCs is that in senile osteoporosis, MSCs undergo cellular senescence resulting in a growth arrest [57].

Finally, it has been demonstrated that MSCs isolated from mice with experimental osteoporosis have a significantly lower anti-inflammatory potential and lower expression levels of immunomodulatory cytokines including TGF-β and IL-10 compared to MSCs derived from control mice [58].

Thus, increasing the viability, as well as the proliferative and osteogenic differentiation capacity, while retaining the anti-inflammatory and immunomodulatory potential of MSCs are still essential steps required to deliver a successful and reliable autologous stem cell-based therapy for osteoporosis.

## Impact of 3D cell culture on bone regeneration-relevant properties of MSCs

Traditional, two-dimensional (2D) cell culture on flat plastic or glass surfaces is still widely used in regenerative medicine and basic stem cell research. However, removing stem cells from their endogenous 3D niche results in unnatural polarity, changes in growth kinetics and last but not least, an altered differentiation potential. In this context, 2D culture of MSCs is known to result in a loss of multipotency and premature cellular senescence [59]. Moreover, 2D cell culture conditions have been linked to accumulation of chromosomal aberrations within the MSC genome [60, 61].

To address these problems, MSCs have been subjected to a wide range of 3D cell culture methods including cultivation as scaffold-free spheroids [62, 63] (reviewed in [64]) and different hydrogel scaffolds including alginates [65], collagen [66], Matrigel [67] and various formulations of cellulose [68–71].

In the following sections, we will summarise current knowledge on the impact of 3D cell culture on fundamental properties of MSCs that are pivotal in bone regeneration. To directly assess the effects of 3D cell culture on MSCs, the following sections only cover studies that have explicitly used 2D controls. Moreover, reports using less than two independent methods to determine the cell fate have been excluded.

### Viability and proliferation

3D cell culture has been widely used in cancer research. Notably, the impact of 3D cultivation on cell viability and proliferation has been shown to be cell type and technique dependent. While some studies reported an increase in viability and proliferation compared to the respective 3D controls, others observed opposite effects (reviewed in [72]). This can be, at least partly, attributed to the nature of the 3D cell culture method. Since osteoporosis and ageing in general negatively affect viability and proliferation of MSCs, assessing the impact of 3D cell culture technology on these parameters is important for developing successful therapeutic strategies.

Briefly, spheroid cell culture has often been associated with decreases in cellular proliferation and viability, while 3D hydrogels can have an equal or superior performance compared to 2D cultures (reviewed in [73]). Contrary to reports on the impact of spheroid culture on other cell types including cancer cells and neural crest-derived stem cells indicating negative effects on cell proliferation and viability [74], the consequences of cultivation of MSCs as spheroids are still controversial. While some studies suggested an increase in cell viability and proliferation of MSCs within the spheroids [75, 76], others reported no effects [77] or a reduced cell proliferation [78–80]. This could be attributed to a non-homogenous nutrient and oxygen supply and impaired waste product diffusion in the core if a critical sphere size has been reached (reviewed in [64]).

Similar to the contradicting reports on the impact of spheroid cultures on cell proliferation and viability, the effects of 3D culture of MSCs on scaffolds and within hydrogels are complex. While some 3D scaffolds including gelatine, poly lactic-co-glycolic acid and chitosan do not seem to affect viability and the proliferation of MSCs [81], other substrates have been reported to change these properties. In this context, collagen seems to have different effects depending on its preparation and the source of MSCs. Lo and colleagues did not observe significant changes in cell viability and proliferation of collagen-embedded human MSCs [81], while rat bone marrow MSCs (BM-MSCs) have been reported to increase their proliferation in the same scaffold [82]. Other reports also suggest that small differences in the scaffold composition and preparation technique can be decisive for the biological outcome.

In 2013, a study by Rampichová et al. suggested that 3D cultivation of minipig MSCs in electrospun 3D polycaprolactone increases both, their proliferation and viability [83].

In contrast to these findings, 3D printed polycaprolactone/tricalcium phosphate did not affect the viability of human adipose tissue-derived MSCs (AD-MSCs) and BM-MSCs [84]. Interestingly, Brennan and colleagues demonstrated that sprayed micro-fibre polycaprolactone scaffolds increase human BM-MSC proliferation, while electrospun variants of the same scaffold decreased proliferation compared to the 2D controls [85]. This provides further evidence that small differences in the nature of the scaffold can dictate very different cell fates. In general accordance with this hypothesis, woven biodegradable composite fibres from poly (l-lactic acid) substituted with hydroxyapatite had an overall positive effect on BM-MSC viability, while the same substrate alone reduced it [86]. Conversely, Nguyen and colleagues reported that electrospun scaffolds significantly reduced proliferation of human MSCs as early as 7 days after seeding on nanofibrous scaffolds composed of poly (l-lactic acid) and type I collagen [87].

In our lab, we have studied the effects of 3D nanofibrillar cellulose on human MSCs derived from various tissue types. In our hands, low concentrations (0.2%) of nanofibrillar cellulose did not negatively affect the proliferation of BM-MSCs, AD-MSCs and palatal MSCs, whereas higher concentrations (0.5%) significantly reduced cellular viability and proliferation compared to 2D controls [70]. In contrast, a follow-up study showed that an anionic form of nanofibrillar cellulose significantly increased the viability of human AD-MSCs [71]. Similarly, Yin and colleagues recently reported that 3D cultivation of AD-MSCs in a nanofibrous polysaccharide hydrogel increases their proliferation and viability [88].

Overall, the effects of 3D seem to rely on multiple factors including but not limited to the nature of the scaffold, its preparation and the origin of the MSCs.

### Osteogenic differentiation

In addition to influencing the viability and proliferation of MSCs, multiple reports suggest that 3D cultivation might have an impact on their osteogenic differentiation. In contrast to the controversial impact of MSC viability, 3D cell culture seems to increase the osteogenic differentiation compared to conventional 2D culture.

It has been demonstrated that cultivation of rat BM-MSCs as spheroids resulted in an increased osteogenic potential compared to 2D controls [67]. This has been evidenced by a higher expression of Osterix, increased ALP activity and higher level of mineralisation in vitro. Similarly, scaffold-free 3D spheroid culture of murine BM-MSCs resulted in a 5-fold higher mineralisation compared to osteogenic differentiation of 2D cultures [79].

A 2008 report provided evidence that 3D nanofibrillar scaffolds prepared from rat tail collagen increased the expression of osteogenic markers including type I collagen, osteopontin (OPN) and osteonectin in human AD-MSCs compared to differentiation conducted in 2D. Although Alizarin Red S staining in this report suggested that calcification could also be increased, the significance of the data was not assessed [89]. Similar effects on the expression levels of osteocalcin, OCN and OPN in rat BM-MSCs were observed if purified collagen was used to create a 3D scaffold [82]. Notably, von Kossa staining revealed statistically significant increase of calcification. In 2012, Nguyen and colleagues used a blended scaffold composed of collagen and electrospun poly(l-lactic acid) to study the influence of 3D cell culture on the differentiation of human MSCs of undefined origin. In their study, they showed that differentiation in 3D significantly increases expression of OCN and OPN and augments the levels of calcification compared to cells differentiated in 2D [87]. Moreover, use of pure poly (l-lactic acid) scaffolds as a substrate showed similar increases in expression of pro-osteogenic markers compared to human BM-MSCs differentiated in 2D [86]. This was accompanied by a significant increase of Ca^2+^ deposition demonstrated via von Kossa and Alizarin Red S staining.

In addition to collagen, poly(l-lactic acid) and blends of these materials, Matrigel, a hydrogel mainly composed of type IV collagens, entactin, perlecan and laminin has been shown to increase the osteogenic differentiation potential compared to 2D cell culture. If differentiated within a Matrigel scaffold, human BM-MSCs showed higher activity of alkaline phosphatase (an early marker of osteogenesis) compared to the 2D counterparts [90]. In addition, Alizarin Red S staining revealed higher Ca^2+^ deposition in cells differentiated in 3D.

As alternative scaffolds, different polycaprolactone-based substrates have been explored in combination with MSCs. An elastic 3D poly (ε-caprolactone) has been shown to increase the expression of bone sialoprotein and OCN in minipig BM-MSCs subjected to differentiation in 3D [83]. In accordance with this report, polycaprolactone-tricalcium phosphate scaffolds and jet sprayed micro-fibre polycaprolactone scaffolds have shown similar osteoinductive effects in human foetal and adult BM-MSCs [85, 91]. There are also reports on increased osteogenic differentiation of AD-MSCs and BM-MSCs in 3D vs 2D, if gelatine and blends of gelatine and alginate are used as a scaffold [81, 92].

### Cellular senescence

Increased cellular senescence of MSCs is one of the contributing factors for poor osteogenic regeneration in osteoporosis. Although studying the impact of 3D cell culture on cellular senescence is a relatively new field, there is some evidence suggesting that propagation of cells in 3D might reduce their cellular senescence. In this context, Cheng and colleagues have clearly demonstrated that 3D cultivation of human AD-MSCs as scaffold-free spheroids prolongs their replicative lifespan and decreases their senescence compared to 2D controls [93]. However, a recent report provided evidence that the number of senescent cells within MSC spheroids increases over time [94].

In contrast, it has been reported that cultivation of human periodontal ligament fibroblasts, a cell type which is phenotypically indistinguishable from MSCs [95], in 3D methylcellulose hydrogels results in suppression of TNF-α-induced cellular senescence and an increase in proliferation and migration [96]. In an attempt to study the stress response in human endometrial MSCs cultivated as 3D spheroids in comparison to conventional 2D culture, Domnina and colleagues exposed cells cultivated under both conditions to heat shock and treatment with H_2_O_2_ [97]. This study demonstrated that 2D monolayer MSCs developed premature senescence, while MSCs in 3D exhibited decreased viability without cell cycle arrest and other signs of senescence. Interestingly, subsequent analyses revealed that 3D MSCs that did not undergo apoptosis in response to heat shock-induced stress, resumed cell division and had comparable proliferation rates and cell cycle dynamics compared to the 2D controls. These results suggest that 3D culture can be helpful in eliminating senescent cells from MSC cultures.

Finally, a very recent comparison of cellular senescence in human adipose-derived MSCs cultivated in a monolayer and cells in a commercial polysaccharide hydrogel revealed significant reduction of senescence-associated β-galactosidase activity in 3D. Moreover, an overall higher telomerase activity and greater telomere length was observed in MSCs in 3D. This was accompanied by a higher adipogenic and osteogenic differentiation potential [88].

Overall, this promising data suggests that 3D cultivation might reduce cellular senescence of MSCs, thereby facilitating the ability to obtain clinically relevant cell numbers in a shorter time frame.

### MSC secretome, immunomodulation and anti-inflammatory potential

In addition to its direct impact on characteristics of MSCs, 3D cell culture has been suggested to modulate the regenerative potential of their secretome.

Although there is some evidence that 3D cultivation as spheroids can reduce the anti-inflammatory and immunomodulatory potential of MSCs [98], most secretomes produced by MSCs cultivated in 3D hydrogels seem to have higher anti-inflammatory and immunomodulatory properties, and an overall greater regenerative potential compared to both spheroids and conventionally cultivated cells.

Cultivation of human AD-MSCs as spheroids has been shown to increase expression of vascular endothelial growth factor (VEGF) and hepatocyte growth factor (HGF) compared to 2D, although no significant differences in the levels of fibroblast growth factor-2 (FGF-2) have been observed [93]. Moreover, human BM-MSCs cultivated as spheroids generated by the hanging drop method showed higher levels of the anti-inflammatory cytokine interleukin 24 (IL-24) [63]. Interestingly, the same study provided evidence that spheroid MSCs were more efficient in supressing LPS-induced inflammation compared to their 2D counterparts. In accordance with these findings, dynamic culture of human MSC spheroids in spinner flasks has been shown to result in increased levels of IL-24 secretion [99]. A more recent study provided further evidence for a change of paracrine properties of spheroid MSCs. Briefly, Sun and colleagues demonstrated that MSC spheroids secrete higher levels of VEGF [100]. In addition, spheroid cultures of MSCs have been demonstrated to produce higher levels of prostaglandin E2 that mediated polarisation of macrophages towards the anti-inflammatory M2 phenotype [101, 102].

In addition to spheroid cultures, a higher regenerative potential of secretomes from 3D cultivated AD-MSCs compared to 2D culture has been reported by Chierchia et al. [103]. In this study, MSC secretome-mediated neuroprotection of SH-SY5Y neuroblastoma cells following 6-hydroxydopamine treatment was increased if the MSCs were cultivated in bovine collagen/polyethylene glycol and collagen/low-molecular weight hyaluronic acid hydrogels. However, to date, no molecular profiling of these secretomes has been reported.

In 2018, it was also reported that 3D spheroid cultures of human BM-MSCs secreted higher levels of VEGF compared to 2D controls [62]. These findings are in line with several other reports demonstrating that 3D cultivation of AD-MSCs results in an increased secretion of multiple growth factors including HGF, VEGF, stromal cell-derived factor **(**SDF) and FGF-2 [75, 104, 105].

More recently, Carter and colleagues studied the wound healing potential of 2D, and 3D cultivated human BM-MSCs in vitro and ex vivo [106]. In their study, they demonstrated that 3D cell culture of MSCs on electrospun gelatine/polycaprolactone fibres increased the secretion of FGF-2, IL-6, VEGF and HGF compared to 2D controls. In addition, 3D cell culture significantly improved secretome-mediated corneal wound healing in a rabbit corneal organ culture system [106].

A comparative analysis of the secretomes of umbilical cord MSCs cultivated as spheroids and as adherent monolayer revealed that 3D cultivation significantly increases the secretion of IL-10, LIF, FGF-2, I-309, SCF and GM-CSF [107]. Moreover, this study also showed that secretome from 3D cultivated MSCs has superior regenerative and anti-inflammatory potential in an in vivo model of arthritis in adult Wister rats.

Overall, 3D cultivation is associated with increased level of secreted molecular factors that are pivotal in bone regeneration (see Table 1). Thus, the findings summarised above suggest that 3D cultivation can indeed increase the levels of bone regeneration-relevant components within the MSC secretome.

**Table 1:** Examples of bone homeostasis-relevant paracrine factors released by MSCs

Target	Paracrine factors	References
Osteogenic differentiation	TGFβ, BMP-1 and miR-196a	[108–110]
Anti-inflammation	IL-10, TGFβ, TSG-6, LIF, miR-146a-5p, miR-548e-5p, let 7 family, miR-145	[43, 108, 111–115]
Immunomodulation	PGE-2, HLA-G5, HGF, iNOS, TGFβ and IL-10	[108, 116, 117]
Anti-apoptosis	VEGF, HGF, IGF-1, stanniocalcin-1, GM-CSF and TGFβ	[108, 118]
Angiogenesis	VEGF, IGF-1, PIGF, MCP-1, FGF-2 and IL-6	[118]
Support of growth and differentiation of stem cells	SCF, LIF, M-CSF, SDF-1 and angiopoietin-1	[113, 118]
Chemoattraction	CCL2, 3, 4, 5, 6, 20 and 26 and CXCL5, 11, 1, 2, 8, 10 and 12	[117, 118]
Anti-fibrosis	HGF, FGF-2 and adrenomedullin	[113, 118, 119]

## Potential mechanisms instructing osteogenic potential of MSCs in 3D

In addition to biochemical signals, mechanophysical cues including surface stiffness and presence of micro- and nanoscale pores, pillars and pits are known to heavily influence the fate of stem cells [120]. In vivo, many of these cues are provided by the extracellular matrix (ECM) and include matrix stiffness and presence of pores, pillars and pit-like structures at microscale and nanoscale (macro- and nanotopography). The stiffness of the ECM in the human body ranges from 0.1–1 kPa in the brain to 25–40 kPa in osteoids (unmineralised, organic portion of the bone matrix that develops prior to the maturation of bone tissue) [121]. In contrast, conventional cell culture plastic material is very rigid with a stiffness of 1–10 GPa [122]. Indeed, MSCs have been described to be highly sensitive to substrate stiffness. However, reports on the optimal osteoinductive stiffness are conflicting. While Hwang et al. reported a stiffness of 4.47 kPa as osteoinductive [95], a much higher Young’s modulus ranging from 62 to 800 kPa has been suggested in studies from other laboratories [94, 96]. Importantly, although stiffness can be manipulated in 2D to study MSC differentiation [121, 123], it is important to consider that in vivo MSCs are exposed to the mechanophysical cues in all three dimensions of the surrounding niche. Therefore, spheroid cultures and hydrogels surrounding the cells might provide stronger inductive cues compared to the more artificial environment in 2D.

In addition to the stiffness, both macro- and nanotopography of the stem cell niche play an inductive role in cell differentiation.

In this context, it has been established that nanopores with a diameter of 30 nm within elastic (collagen) and rigid 2D surfaces (titanium) are strong osteoinductive cues for adult stem cells [124–126]. In addition, 2D micropores of 60 and 550 μm have been reported to have a positive impact on osteogenic differentiation [127]. Interestingly, electron microscopic analyses of the structure of various hydrogels including different forms of nanofibrillar cellulose and fibrin have revealed a presence both nano- and micropores [70, 71, 128]. Thus, these nanoscopic and microscopic features could provide a potential explanation for the increased osteogenic potential of MSCs in 3D. Notably, differences in the nano-, micro- and macropore profiles of different scaffolds would have an impact on nutrient, oxygen and waste product diffusion and could explain the differences in viability and proliferation described earlier.

Interestingly, physical properties of scaffolds can also dictate the paracrine potential of MSCs [129] with substrates with an elasticity of 20 kPa resulting in an increase of expression of anti-inflammatory factors compared to more elastic substrates with an elasticity of 2 kPa. In 3D and depending on the nature of the hydrogel, retention of the secreted soluble factors and EVs could also contribute to the increased osteogenic potential. Indeed, peptide-modified hyaluronic acid hydrogels have been shown to retain EVs and show beneficial therapeutic outcomes in animal models of spinal cord injury [130]. Similarly, umbilical cord MSC-derived EVs encapsulated in functional peptide hydrogels increased the retention and stability of exosomes and improved heart function in a rat myocardial infarction model [131]. Recently, it has been demonstrated that alginate hydrogels loaded with AD-MSC-derived EVs can promote tissue regeneration in full-thickness skin wounds [132]. Finally, cellulose hydrogels have been shown to retain EVs and allow their continuous release from encapsulated cells [133]. Therefore, a potential additional mechanism increasing the osteogenic potential MSCs in 3D hydrogels could be a retention of soluble pro-osteogenic factors and EVs. This could exhibit both autocrine on and paracrine effects that drive osteogenesis in 3D.

## Conclusions

**Fig. 3:**
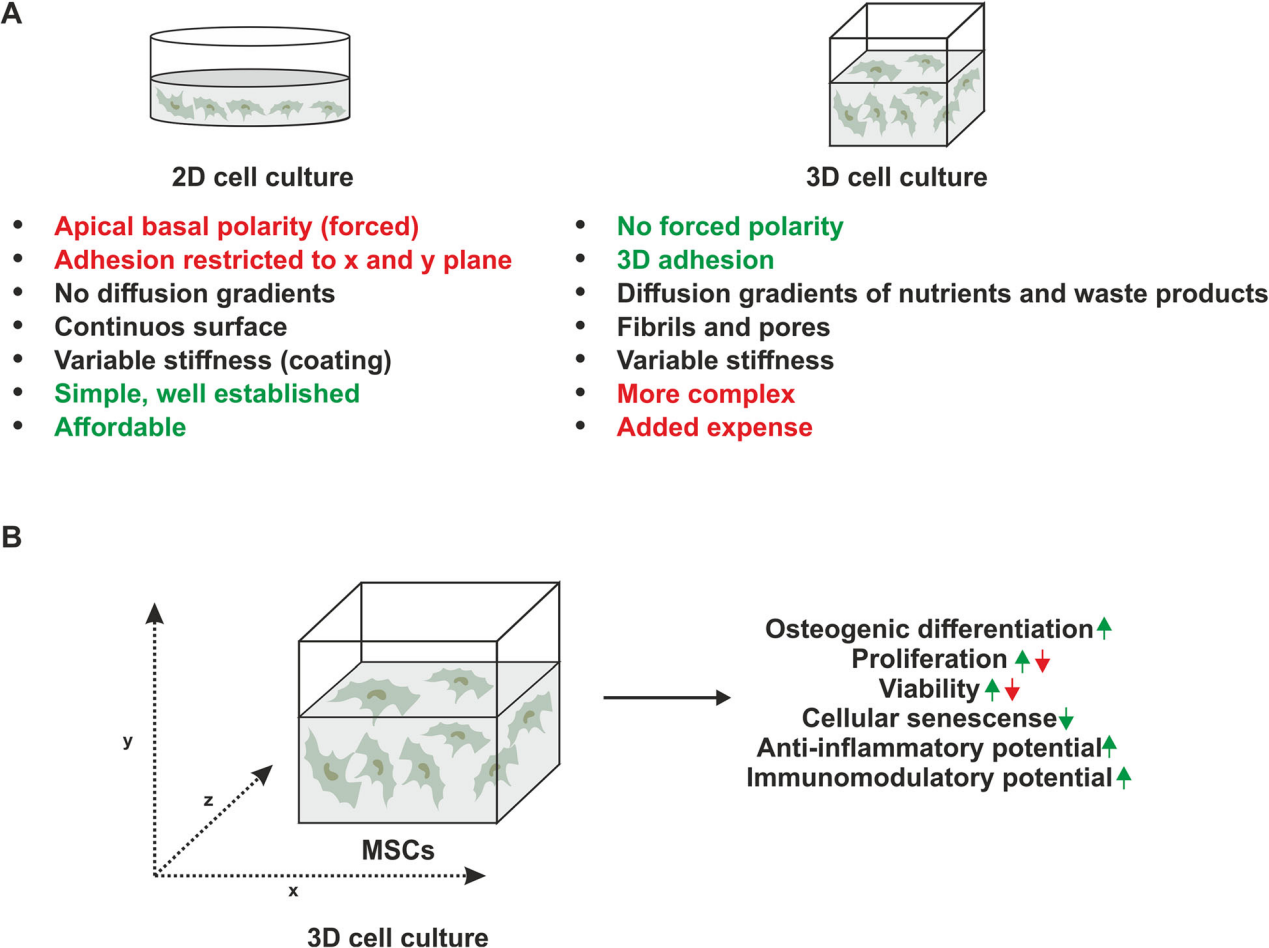
Effects of 3D cell culture on MSC-properties relevant for bone regeneration. **a** Advantages and drawbacks of 3D cell culture compared to traditional 2D cell cultivation. Advantages are highlighted in green, drawbacks in red. **b** 3D cell culture increases osteogenic differentiation potential of MSCs and decreases their cellular senescence. In addition, both anti-inflammatory and immunomodulatory potential of MSCs seems to be increased under 3D conditions. In contrast, the effects of 3D cell culture on cell viability and proliferation are dependent on the nature of the 3D cell culture and the cell density

As outlined above, the nature of the 3D scaffolds and small variations in their composition can influence the viability as well as the osteogenic and paracrine potential of MSCs (Fig. 3). Therefore, rational design of 3D cell culture parameters could further improve the performance of MCSs in bone regeneration. To achieve this, an ideal 3D scaffold should be biocompatible and osteo-conductive-inductive (reviewed in [134]). Moreover, scaffold stiffness, its topography and potential retention and release of paracrine factors need to be considered. Finally, as electrical stimulation of MSCs has been shown to enhance the osteogenic potential of MSCs [135], conductivity could also represent an important factor in designing optimal osteoinductive biomaterials.

## Data Availability

Not applicable.

## Abbreviations

2D: Two-dimensional
3D: Three-dimensional
AD-MSCs: Adipose tissue-derived MSCs
ALP: Alkaline phosphatase
BM-MSCs: Bone marrow MSCs
BMP-1: Bone morphogenetic protein 1
BPs: Bisphosphonates
CCL: C-C motif chemokine ligand
CXCL: C-X-C motif chemokine ligand
EVs: Extracellular vesicles
FGF-2: Basic fibroblast growth factor
HGF: Hepatocyte growth factor
HLA-G5: Histocompatibility antigen, class I, G5
HM-CSF: Granulocyte-macrophage colony-stimulating factor
HRT: Hormone replacement therapy
IFNγ: Interferon γ
IGF-1: Insulin-like growth factor 1
IL-10: Interleukin-10
IL-24: Interleukin-24
IL-1β: Interleukin-1β
IL-6: Interleukin-6
iNOS: Inducible nitric oxide synthase
LIF: Leukaemia inhibitory factor
MCP-1: Monocyte chemoattractant protein-1
M-CSF: Macrophage colony-stimulating factor
MSCs: Mesenchymal stromal cells/mesenchymal stem cells
OCN: Osteocalcin
OPN: Osteopontin
PGE-2: Prostaglandin E2
PlGF: Placental growth factor
PTH: Parathyroid hormone therapy
RANKL/M-CSF: Receptor activator of nuclear factor kappa-Β ligand/macrophage colony-stimulating factor
RANKL: Receptor activator of nuclear factor kappa-Β ligand
SCF: Stem cell factor
SDF-1: Stromal cell-derived factor 1
SERMs: Selective oestrogen-receptor modulators
SOST: Sclerostin
TGF-β: Tumour growth factor-β
TNF-α: Tumour necrosis factor-α
TSG-6: TNF-α-stimulated gene-6
VEGF: Vascular endothelial growth factor
